# Vital Capacity

**Published:** 1878-10

**Authors:** 


### VITAL CAPACITY.

To illustrate: if a man is five feet seven inches high, his healthful "vital capacity" is two hundred and twenty-two cubic inches; (three 2's, easy of remembrance;) if he measures five feet eight inches, then his "vital capacity" must be eight cubic inches of air more; that is, two hundred and thirty. Some of these statements were made in the October number, under the head of "Spirometry;" the spirometer being the instrument for "measuring the breath," that is, the "vital capacity" of any individual; and they are here repeated, in order to make a connected but more practical application of this doctrine of "vital capacity." It would seem, that the amount of a man's lungs was determined by the development of his chest, by the girth around it; but the hard fact is, that it is not the case. Of two men of equal age and hight, one measuring two feet and a half, and the other three feet and a half or four feet, the large chest will not have a greater "vital capacity," will not deliver, at one full expiration, one cubic inch more of air than the smaller-waisted man. This fact is repeated, is constantly coming under the writer's observation.

When it is said that a man five feet seven inches high will, if his lungs are sound and are working fully and well, deliver two hundred and twenty-two cubic inches of air at one full expiration, or take in that amount at one full inspiration, it is not meant to say that he takes that much air into the lungs at each ordinary inspiration, for he really takes in much less, probably a pint, or some forty cubic inches; but the proportions are the same between a full breath, whatever that may be, and the ordinary inspiration of each.

In connection with that wide-spreading malady, "Consumption," and the great fact that as that disease progresses the breath becomes shorter — that is, the "vital capacity" becomes less; taking into account, also, that consumption never begins its actual inroads on the lungs until the "vital capacity" has for weeks and months been less than the healthful standard, and that consumption can not exist without a large diminution of the "vital capacity," the great practical fact comes up before the mind with striking importance, that the very first thing to be determined, in any case of existing or apprehended consumption, is, "What is the vital capacity?" or, "What is the actual capacity of the lungs for receiving air?" Then, if it is found to be below the healthful standard, there is cause for alarm, and measures should be taken to increase this "actual" capacity of the lungs; and those measures should not be intermitted for a single day, until the desired end is attained, *with a margin.*

It is certainly gratifying to know that the decaying power of the lungs can be re-developed; physicians of various schools have succeeded in devising means, according to the principles of their particular creed. Each one may think his own the best. Our method has advantages not to be lightly valued; it does not cost a dollar of money, and consequently is available to every sufferer, however poor; it does not confine to the house; it is practicable to all; it is attended with no pain; it is combined with no mystery; it is plain to the commonest understanding; and the improvement of the patient, or the fatal progress of the disease, is so certainly marked, that neither the patient nor the physician can be deceived under any ordinary circumstances. But there is an objection to it, which is fatal as to its good effects in perhaps nine cases out of ten. Not one person in ten has the moral power, has force of character enough to carry it out. It would do more or less good in every stage of consumptive disease; can never do any injury whatever; but it is so much easier to drink whisky; to swallow cod-liver oil; to swill porter, ale and beer; to purchase cough-drops and expectorants; there is such a preference for alleviants over eradicators, that it is hardly worth the trouble to explain the philosophy of it to one in ten. Yet, by it, or its substitutes, *as a main means,* persons have attained better health, and lived in considerable comfort for two, ten, and twenty years afterward; and the same results must occur in all time to come, for *nature's agencies never lose their power.* It is by the aid of the foregoing principles, together with observations made daily on that class of diseases to which spirometry, or the doctrine of "vital capacity," is applicable, that the following results have been attained:

1st. Persons who have been abandoned to die of consumption have been ascertained not to have that disease, and, as a consequence, are living at this day.

2d. Others who were not considered to have had that malady, have nevertheless applied for an examination and opinion, and have been found to be in the last stages of that dreaded ailment.

3d. The first spirometer ever made for sale in this country was made to the order of the writer some fifteen years ago, and *no case has ever come to his knowledge which, having been pronounced hopeless by him, has ever recovered.*

There are too many who claim to have special experience and ability in diseases of the air-passages, who pronounce of every man who calls, without exception, "Yours is a very bad case;" and on being appealed to, to know to what extent restoration can be reasonably expected, they reply almost invariably, and in the most decided and confident terms: "I have cured worse cases than yours, and can cure you, with the utmost certainty." The course of the true physician is widely different; both his honor and a common humanity imperatively call upon him to pronounce a plain, candid, and unequivocal opinion. If a hopeless case is recklessly declared "curable," time will prove the falsehood. If a man is laboring under the impression that he has an incurable disease, when on examination it is clear there is no approach to it, it is a cruelty and a robbery to keep him under the false impression, for the purpose of working on his fears, and detaining him from home, under heavy expenses, for the alone object of making a heavy bill.

On the other hand, the cruelty and the robbery are equally vile, if, when the patient is known to be in a hopeless condition, he is detained with promises of cure week after week, and at a heavy, or to him and his, a ruinous expense, until return to home and friends is impossible.

If a man is not consumptive, and is plainly told so, such a burden is sometimes taken from his mind, that a new life is in fused into him; he rises above the depressions which were crushing him into the grave, throws off disease, and goes forth in a few days a new being and a well man.

On the other hand, if the symptoms are really grave, it is better that the patient should know it, than be allowed to consider them slight; for then he will be prevented from making those exertions for recovery which are indispensable to his safety. No man will work for life if he is assured that life is not in danger.

Last year I was called to see the wife of a New-York merchant. The husband had been informed by his family physician that he had no hope of her recovery; that she was in a decline. No one but a physician can know how closely he is watched, from the instant of his entering a house in which a patient lies, on whom he is expected to look, and to decide the momentous question of life or death. How the servants usher him into the bed-chamber with noiseless step and deferential speech! How the children, with mouth agape and open eyes, look into the very soul of the medical man, so loudly mute, so beseechingly, as if the power of life and death over their mother was in his keeping! How the husband, with compressed lips and concealed emotion, stands on one side, and under the guise of no observation, reads every gesture made, every look given, weighs every word uttered; determines the bearing of every inquiry made and question answered! These are ordeals through which the city physician is constantly called to pass; and fortunate is he who has perfect control over the expressions of the usually tell-tale countenance, and withal has the grand, supporting influence afforded by a consciousness of the ability to stand in his situation and fill it; and also that other consciousness of an ability of truthfulness under the conviction that deception can be of no permanent or ultimate good. After examining the merchant's wife, with the care which it seemed to merit, and with the earnest desire to make no mistake, it was concluded to say, in the very face of the opinion of the attendant physician: "I really do not think much is the matter with you." In ten days she had made a journey to the National Capital, and spent a fortnight thereafter in seeing its sights. When she returned home, she had no special need of any medical advice.

In passing an opinion as to the nature of any given case of consumptive disease, it is so much less troublesome to speak truly; is such a relief to be free from the incubus of a falsehood; the incubus of always being on guard against belying one's self, that it is wonderful that any man can be found who is willing to set himself deliberately to the task of uttering an untruth and sustaining it afterward for weeks and even months. Nothing can be adequate to such an attempt, but a greed of gold so desperate and mean, as to have eaten out every exalted principle of our nature.

Not long ago an only son was brought for an examination.

It was clear that the youth must die within a month, and that nothing could be done which would be equal to the advantages of being at home in his father's house, and under a mother's care. Any reasonable and even an unreasonable weekly charge would have been more than willingly paid, if even an implied promise of material benefit could have been extracted; but one always sleeps better under a consciousness of truthfulness, with ten dollars in his pocket, than with the conviction that he is a mean misleader, with fifty dollars in his "bag."

Then, again, there is a perfectly delightful feeling which comes over a man (every time he thinks of it) in the felt conviction that he is believed in. It is always a sad thing, and a hard, hard task, to be compelled to say to a doting parent: "Your child can not recover under any conceivable circumstances." But pay comes afterward; when the child has been dead for years, and time has soothed the sorrow over his death, the compensation comes in the saying to this neighbor and that friend and the other acquaintance: "The Doctor did not deceive me." In the case above, the parents were advised to return home without delay, as it was probable their child would not live over a month. The family physician pronounced the opinion incredible, and gave strong assurances of his conviction that he would be well in a month. He was well—in the grave!

It is reasonable to suppose, when a physician is called upon to give his candid opinion of a case, that the family and friends would feel themselves under considerable obligations when such an opinion was given, although unfavorable, especially when that opinion was subsequently found to have been literally correct. But this is not always the case.

I was called last year to see a lady of about sixty years of age. All the appointments of the mansion indicated wealth, position, and culture. After the examination, and retiring from the sick-chamber, I intimated to the friends that the case was a very grave one; that I did not think it was worth while for me to prescribe for her, and that she had better remain under the care of the family physician. I was then pressed to know how long I thought she might live. They seemed incredulous at my reply, not having regarded her case so serious as I seemed to do. As she possessed considerable property, and I considered it of importance so to express myself as not to be misunderstood, I said to them: "Nothing short of the power that made her can keep her alive ten days." She died within the time. The family have shown their non-appreciation of a candid opinion ever since.

The intelligent reader will want to know how is it that a physician can tell with such certainty what will be the result in cases like the above. In the first place, over twenty years' special attention to these ailments would give the dullest man facilities of discrimination.

Again, sometimes men can do things without being able to tell how—Barnum's lightning arithmetical calculator for example, and others of that class. I have many times formed an opinion of the case of a stranger entering my office, before he has had time to take his seat, and a painfully minute examination has but confirmed the original opinion. When a man is in the last stages of consumption, there is an indefinable something pervading the whole person, combined in action, speech, gesture, intonation, and expression of countenance, which, although hard to be put in words, tells an "o'er true tale."

A man who is in the decided stages of consumption has not one but several symptoms, each one of which is in itself alarming, but all combined, make an erroneous opinion in a measure impossible.

A certain set of symptons may exist without the physician being able to say positively, "You have consumption;" if such an one has a healthful "vital capacity," it is certain that it can not be consumption. Another man may not have as many of these bad symptoms, or none of them may be so aggravated, by reason of constitution, temperament, duration, etc., yet, if the spirometer shows that he is deficient in "vital capacity," then the existence of consumptive disease becomes a demonstration.

When I have ascertained that a man has diminished "vital capacity;" that his pulse is much too fast at any and all hours; that he has been losing in flesh and strength and breath, expressed by the complaint of "great shortness of breath;" that, on placing the ear on the chest under the collar-bone, it gives no more sound than if it were laid upon a dead wall; or that it gives such a sound as is made by blowing into a large-mouthed vial; or that there is the sound of blowing through a tube into a vessel of thick soap-suds, I know that consumption is present in the form of the presence of tubercles, fatal in their numbers; or in the form of a dry cavity in the lungs, showing that they have been eaten away; or a partially filled cavity, indicating that the lungs are in an actual state of decay; of consuming, or consumption; and when such is the case, no honest physician can hesitate to declare that death will most likely be the result; for when the lungs once begin to decay, giving wasting of flesh, strength, and health, the issue is fatal in almost every one of a thousand cases.

But suppose all the above symptoms exist, except that the sound given out is like the twittering of many little birds, then it is not only not consumption, but it is next to impossible, in ninety-nine cases out of a hundred, that the person will ever have consumption. And why? simply because the bird-like twittering heard, when the ear is laid flat on the chest, was never known to be given out by a consumptive patient, but is always given out by an asthmatic; and asthmatics seldom die of consumption, or of any thing else except of old age; in a sense, they die daily; suffer a thousand deaths, but wheeze on, until they dry up to skin and bone, or become dropsical.

But suppose all the symptoms enumerated awhile ago were present, except the twittering sound and a quick pulse, with a "tremendous cough" added, liable to come on any hour of the night or day, then it is clear that it is neither consumption nor asthma, but common chronic bronchitis, and the man has a good chance of living to the age of sixty or seventy years.

It will be observed that I have not enumerated cough and night-sweats as symptoms of consumption; this is simply because they are not always present. "The books" give cases where persons were never observed to have had any cough; and yet, when examined after death, the condition of the lungs showed that consumption was the sole cause of death. "Night-sweats" are always alarming; this has arisen from the fact that persons in the last stages of consumption frequently have them. But this is not always the case; and, again, "night-sweats" are common to several debilitating diseases; sometimes they arise from an anxious state of the mind; at others from accidental circumstances, such as a great change to warmer weather; an over-amount of bed-clothing, or an over-heated room.

